# Supplementary material for: Comparative Fluorescence In Situ Hybridization (FISH) Mapping of Twenty-Three Endogenous Jaagsiekte Sheep Retrovirus (enJSRVs) in Sheep (Ovis aries) and River Buffalo (Bubalus bubalis) Chromosomes
Source: Animals (Basel). 2022 Oct 19;12(20):2834. doi: 10.3390/ani12202834 (PMC9597706; doi:10.3390/ani12202834)
Supplement: Supplementary file 1 [file animals-12-02834-s001.zip › animals-1911534-supplementary.pdf]

Table S1. List of genes present in the ORA6q13 and included between QRFPR and TRAM1L1 (from: https://www.ncbi.nlm.nih.gov/genome/gdv/browser/genome/?id=GCF_016772045.1), their main functions and references.

GENE SYMBOL	GENE NAME	MAIN FUNCTIONS	REFERENCES
**QRFPR**	Pyroglutamylated RFamide Peptide Receptor	-It acts as important role in neurodevelopment<br>-Functional studies have shown that, in mammals, the 26RFa/QRFP-QRFPR system may regulate various functions, including food intake, energy homeostasis, bone formation, pituitary hormone secretion, steroidogenesis, nociceptive transmission, and blood pressure	[1<br>[2]
**TNIP3**	TNFAIP3 interacting protein 3	It is an anti-inflammatory protein that plays an important part in both immune responses and cell death.	[3]
**NDNF**	Neuron Derived Neurotrophic Factor	-It inhibits the migration and invasion of human renal cancer cells through epithelial-mesenchymal transition<br>-functions as a muscle-derived secreted factor, also known as myokine, which exerts protective actions on endothelial cell and cardiomyocyte function	[4]<br>[5]
**PRDM5**	PR/SET Domain 5	It participates in modulate cellular processes, including cell growth, differentiation and apoptosis and as tumor suppressor in different types of cancer.	[6]
**MAD2L1**	mitotic arrest deficient 2 like 1	promotes tumor formation by inducing chromosomal instability and aneuploidy in cells	[7]
**PDE5A**	phosphodiesterase 5A	suppresses proteasome activity, which results in ER stress and subsequent insulin resistance in C2C12 myotubes.	[8]
**FABP2**	fatty acid binding protein 2	It is responsible for the transport of free fatty acids in the intestinal endothelium cells, playing a role in the development and progression of chronic kidney disease	[9]
**USP53**	ubiquitin specific peptidase 53	-regulates osteoblast versus adipocyte lineage commitment<br>-promotes apoptosis and inhibits glycolysis in lung adenocarcinoma through FKBP51-AKT1 signaling	[10]<br>[11]
**MYOZ2**	myozenin 2	-plays an important role in the occurrence and development of gastric cancer<br>-is a novel gene for human hypertrophic cardiomyopathy	[12]<br>[13]
**SYNPO2**	synaptopodin 2	-recessive mutations in this gene indicates it as candidate of monogenic nephrotic syndrome	[14]
**TRNAE-UUC**	transfer RNA glutamic acid (anticodon UUC)	role in the anticodon stem loop in context of codon-anticodon interactions and frame shift mutations	[15]
**TRNAS-GGA**	transfer RNA serine (anticodon GGA)	It's one of genes which can influence the formation of exterior features and productive qualities of sheep	[16]
**SEC24D**	SEC24 homolog D, COPII coat complex component	-Antiviral gene for hepatitis B infection<br>-Strong candidate gene for cartilage maintenance diseases and craniofacial birth defects.<br>-Autosomal recessive osteogenesis imperfecta	[17]<br>[18]<br>[19]

**METTL14**	methyltransferase 14, N6-adenosine-methyltransferase subunit	-Suppresses proliferation and metastasis of colorectal cancer<br>-Induces endothelial cell inflammatory response as well as atherosclerotic plaque formation	[20]<br>[21]
**PRSS12**	serine protease 12	-It plays a significant role in cognitive function and the development of the brain being related to mental retardation	[22]
**NDST3**	N-deacetylase and N-sulfotransferase 3	It's a potent regulator of lysosomal functions and could be targeted to modulate microtubule and lysosomal functions in relevant diseases.	[23]
**TRNAM-CAU**	transfer RNA methionine (anticodon CAU	Mutations in the anticodon region of other tRNAs can overcome specific tRNA deficiencies.	[24]
**TRAM1L1**	translocation associated membrane protein 1 like 1	Putative modulator of testicular 171 steroidogenesis, playing an important role in reproduction	[25]

References of table 3

1. Li, H.; Lou, R.; Xu, X.; Xu, C.; Yu, Y.; Xu Y.; Hu, L.; Xiang, Y.;Lin, X.; Tang, S. The variations in human orphan G protein-coupled receptor QRFPR affect PI3K-AKT-mTOR signaling. *J. Clin. Lab. Anal.* **2021**, Jul, 35(7), e23822.
2. Ukena, K.; Osugi, T.; Leprince, J.; Vaudry, H.; Tsutsui, K. Molecular evolution and function of 26RFa/QRFP and its cognate receptor. J. Mol. Endocrinol. 2014, 52, T119-T131.
3. Zhou, J.; Hu, M.; He, M.; Wang, X.; Sun, D.; Huang, Y.; et al. TNFAIP3 Interacting Protein 3 Is an Activator of Hippo-YAP Signaling Protecting Against Hepatic Ischemia/Reperfusion Injury. *Hepatology* **2021**, Oct, 74(4), 2133-2153.
4. Xia, L.; Li, S.; Liu, Y.; Huang, Y.; Ni, B.; Wan, L.; Mei, H.; Li, X.; Cai, Z.; Li.Z.. NDNF inhibits the migration and invasion of human renal cancer cells through epithelial-mesenchymal transition. *Oncol. Lett*., **2019**, Mar,17(3), 2969-2975.
5. Ozaki, Y.; Ohashi, K.; Otaka, N.; Ogawa, H.; Kawanishi, H.; Neuron-derived neurotrophic factor protects against dexamethasone-induced skeletal muscle atrophy. Biochem. Biophys. *Res. Commun*. **2022**, Feb 19, 593, 5-12.
6. Wang, X.; Chang, H.; Gao, G.; Su, B.; Deng, Q.; Zhou, H.; Wang, Q.; Lin, Y.; Ding Y.. Silencing of PRDM5 increases cell proliferation and inhibits cell apoptosis in glioma. *Int. J. Neurosci.* **2021**, Feb, 131(2):144-153.
7. Zhou, P.; Chen, X.; Li, M.; Sun, X.; Tan, J.; Wang, X.; et al.. Overexpression of PRDM5 promotes acute myeloid leukemia cell proliferation and migration by activating the JNK pathway. *Cancer Med.* **2019**, Jul, 8(8), 3905-3917.
8. Liu, W.; Tian, X.; Wu, T.; Liu, L.; Guo, Y.; Wang, C..PDE5A Suppresses Proteasome Activity Leading to Insulin Resistance in C2C12 Myotubes. *Int. J. Endocrinol.* **2019**, Jan 15, 3054820.
9 Tsai, I.T.; Wu, C.C.; Hung, W.C.; Lee, T.L.; Hsuan, C.F.; et al.; FABP1 and FABP2 as markers of diabetic nephropathy. Observational Study. *Int. J. Med. Sci.* **2020**, Aug 27, 17(15), 2338-2345.
10. Hariri, H.; Addison, W.N.; St-Arnaud, R.. Ubiquitin specific peptidase Usp53 regulates osteoblast versus adipocyte lineage commitment. *Sci. Rep.* **2021**, Apr 19, 11(1), 8418.
11. Zhao, X.; Wu, X.; Wang, H.; Yu, H.; Wang, J. USP53 promotes apoptosis and inhibits glycolysis in lung adenocarcinoma through FKBP51-AKT1 signaling. *Mol. Carcinog.* **2020**, Aug, 59(8), 1000-1011.
12. Li, Y.F.; Zhu, G.Y.; Ma, Y.; Qu, H.Y.. Expression and prognosis of MYOZ2 in gastric cancer. *Eur. Rev. Med. Pharmacol. Sci*. **2018**, Sep 22(18), 5920-5927.
13 Osio, A.; Tan, L.; Chen, S.N.; Lombardi, R.; Nagueh, S.F.; Shete, S.; Roberts, R.; Willerson, J.T.; Marian, A.J. Myozenin 2 is a novel gene for human hypertrophic cardiomyopathy. *Circ. Res*. **2007**, Mar 30, 100(6):766-8.
14. Mao, Y.; Schneider, R.; van der Ven, P.F.M.; Assent, M.; et al.; Recessive Mutations in SYNPO2 as a Candidate of Monogenic Nephrotic Syndrome Kidney. *Int. Rep.* **2020**, Nov 10, 6(2), 472-483.
15. Fandilolu, P.M.; Kamble, A.S.; Sambhare, S.B.; Sonawane, K.D. Conformational preferences and structural analysis of hypermodified nucleoside, peroxywybutosine (o2yW) found at 37th position in anticodon loop of

tRNAPhe and its role in modulating UUC codon-anticodon interactions. *Gene*, **2018**, Jan 30, 641, 310-325. doi: 10.1016/j.gene.2017.10.072. Epub 2017 Oct 26.

16. Krivoruchko, A.Y.; Yatsyk. O.A.; Safaryan, E.Y. Candidate genes for productivity identified by genome-wide association study with indicators of class in the Russian meat merino sheep breed. *Vavilovskii Zhurnal Genet Selektsii*, **2020**, 24(8), 836-843. doi: 10.18699/VJ20.681.
17. Jiang, X.; Zhang, B.; Zhao, J.; Xu, Y.; Han, H.; et al.; Identification and characterization of SEC24D as a susceptibility gene for hepatitis B virus infection. *Sci. Rep*. **2019**, Sep 17, 9(1), 13425.
18. Sarmah, S.; Barrallo-Gimeno, A.; Melville, D.B.; Topczewski, J.; Solnica-Krezel, L.; Knapik, E.W. Sec24D-dependent transport of extracellular matrix proteins is required for zebrafish skeletal morphogenesis. *PLoS One* **2010**, Apr 28, 5(4), e10367.
19. Zhang, H.; Yue, H.; Wang, C.; Gu, J.; He, J.; Fu, W.; Hu, W.; Zhang, Z.. Novel mutations in the SEC24D gene in Chinese families with autosomal recessive osteogenesis imperfecta. Case Reports. *Osteoporos. Int.* **2017** Apr 28(4), 1473-1480.
20. Yang, X.; Zhang, S.; He, C.; Xue, P.; Zhang, L.; et al.; METTL14 suppresses proliferation and metastasis of colorectal cancer by down-regulating oncogenic long non-coding RNA XIST. *Mol. Cancer.* **2020** Feb 28; 19(1), 46.
21. Jian, D.; Wang, Y.; Jian, L.; Tang, H.; Rao, L.; et al. METTL14 aggravates endothelial inflammation and atherosclerosis by increasing FOXO1 N6-methyladeosine modifications. *Theranostics* **2020**, Jul 11, 10(20), 8939-8956.
22. Mitsui, S.; Hidaka, C.; Furihata, M.; Osako, Y.; Yuri, K. A mental retardation gene, motopsin/prss12, modulates cell morphology by interaction with seizure-related gene 6. *Biochem. Biophys. Res. Commun.* **2013**, Jul 12, 436(4), 638-44.
23. Tang, Q.; Liu, M.; Liu, Y.; Hwang, R.D.; Zhang, T.; Wang, J.. NDST3 deacetylates α-tubulin and suppresses V-ATPase assembly and lysosomal acidification. *EMBO J.* **2021**, Oct 1, 40(19), e107204.
24. Deparis, Q.; Duitama, J.; Foulquié-Moreno, M.R.; Thevelein, J.M. Whole-Genome Transformation Promotes tRNA Anticodon Suppressor Mutations under Stress. *mBio*, **2021**, Mar 23, 12(2), e03649-20. doi: 10.1128/mBio.03649-20.
25. Patel, S.K.; Singh, S.K. Pyroglutamylated RFamide peptide 43: A putative modulator of testicular steroidogenesis. *Andrology,* **2020**, 8, 1815–1823.
